# Comparative phylomitogenomic analyses provide insights into adaptation and carcinization in Anomura

**DOI:** 10.1080/19768354.2025.2607863

**Published:** 2026-01-12

**Authors:** Hee-seung Hwang, Jibom Jung

**Affiliations:** a Scripps Institution of Oceanography, University of California San Diego, La Jolla, CA, USA; b School of Biological Sciences and Institute of Biodiversity, Seoul National University, Seoul, South Korea; c Institute for Data Innovation in Science, Seoul National University, Seoul, South Korea

**Keywords:** Gibbs free energy, evolution, Decapoda, marine, mitochondrial genome

## Abstract

Anomura is a morphologically and ecologically diverse infraorder of decapod crustaceans, yet its evolutionary and phylogenetic patterns remain underexplored using mitochondrial genome-based approaches, particularly regarding adaptive evolution across diverse environments. Here, we present a comprehensive phylogenomic analysis of 42 anomuran mitochondrial genomes, including three newly sequenced species: two intertidal Hapalogastrinae (*Hapalogaster dentata* and *Oedignathus inermis*) and one deep-sea pagurid (*Pagurus rathbuni*). The arrangement of protein-coding genes was identical to that reported in previously studied Lithodidae and Paguridae species; however, several tRNA genes exhibited translocations. Moreover, more than half of the 22 tRNA genes were predicted to adopt atypical cloverleaf form, and all protein-coding genes were under purifying selection. In addition, analysis of the mitochondrial control region revealed a conserved repeat structure (∼47 bp motif repeated ∼3.6 times), from which depth-associated Gibbs free energy patterns were broadly inferred. These patterns suggest potential links between control-region stability, depth-dependent adaptation, and the evolutionary process of carcinization. Time-calibrated analyses suggest that *H. dentata* and *O. inermis* diverged from other lithodids approximately 37–50 million years ago, while *P. rathbuni* diverged around 32 million years ago. These divergence events coincide with the Eocene–Oligocene transition, a period characterized by global cooling, sea-level decline, and shifts in ocean circulation. This temporal correspondence suggests that such environmental changes may have been associated with the diversification and adaptive evolution of Anomura. Overall, this study advances our understanding of anomuran phylogeny and highlights the complex interplay among adaptation to the environment, carcinization, and mitochondrial genome evolution.

## Introduction

Anomura (order Decapoda) is a diverse group of crustaceans that inhabit a wide range of marine environments, from intertidal zones to deep-sea hydrothermal vents. Notably, members of the families Aeglidae and Coenobitidae have successfully colonized freshwater and terrestrial habitats, highlighting the high adaptive capacity of anomuran lineages (Tudge et al. 2012; Bracken-Grissom et al. 2013). Morphologically, anomuran crustacean can be broadly categorized into three major body forms: (1) the hermit crab form, characterized by a well-developed abdomen (e.g. Paguridae); (2) the lobster-like form, with a shortened abdomen (e.g. Kiwaidae; yeti crab); and (3) the crab-like form, with a flattened abdomen (e.g. Lithodidae; king crab) (McLaughlin and Lemaitre 1997; Bracken-Grissom et al. 2013). This morphological diversity reflects the evolutionary process of carcinization, a form of convergent evolution in which crustaceans independently evolve a crab-like body plan characterized by a broad cephalothorax, a reduced and ventrally flexed abdomen, and enhanced calcification. In Anomura, carcinization has occurred multiple times independently across different lineages (Morrison et al. 2002; Ahyong and O’Meally 2004; Tsang et al. 2011; Bracken-Grissom et al. 2013).

Due to their morphological and ecological diversity, anomurans have long served as a valuable model for phylogenetic and evolutionary research. While previous research has examined inter-family relationships using nuclear genomic and morphological data, mitochondrial genome-based analyses remain limited in taxonomic scope, with particularly few studies addressing the control region in detail. As a result, conflicting phylogenetic relationships have been reported among major families such as Coenobitidae, Diogenidae, Paguridae, and Lithodidae (Tan et al. 2018; Wang et al. 2019). Furthermore, the molecular basis of adaptations to extreme environments such as intertidal zones, deep-sea habitats, and hydrothermal vents remains poorly understood.

The mitochondrial genome, comprising 13 protein-coding genes, 22 tRNA genes, 2 rRNA genes, and a non-coding control region (Rahman et al. 2019), plays a central role in energy metabolism and is widely used in evolutionary biology (Gissi et al. 2008; Duchene et al. 2012; Miya and Nishida 2015). The control region, in particular, regulates transcription and replication (Shadel and Clayton 1997) and may provide insight into depth-related adaptations and rapid environmental responses. Lower Gibbs free energy (*ΔG*) values in this region often indicate more stable DNA secondary structures, potentially associated with enhanced gene expression under harsh environmental conditions (Clayton 1991; Seibel and Drazen 2007). Despite its ecological significance, few studies have quantitatively examined the relationship between control region architecture, *ΔG* variation, and environmental factors in anomurans.

In this study, we analyzed the mitochondrial genomes of 42 anomuran species, including 39 previously published and three newly sequenced taxa. Our goals were to (1) refine phylogenetic relationships within Anomura, (2) test the carcinization hypothesis, and (3) evaluate possible links between control-region structure and adaptive evolution. Of particular interest are two intertidal hapalogastrins (*Hapalogaster dentata*, *Oedignathus inermis*), noted for their transitional form between pagurids and king crabs, and one deep-sea hermit crab (*Pagurus rathbuni)*. We aim to conduct a comprehensive phylogenetic analysis using all currently available mitochondrial genome data and to perform detailed investigations of each genome to identify key insights into the evolutionary history of Anomura.

## Methods

### NGS sequencing, De Novo assembly, and mitochondrial genome annotation

Genomic DNA was extracted from the first or second ambulatory legs of adult *H. dentata*, *O. inermis*, and *P. rathbuni* specimens, all preserved in 95% ethanol. DNA extraction followed the phenol–chloroform protocol described by Gautam (2022). Voucher specimens were deposited at the National Institute of Biological Resources (NIBR), Korea, under the accession numbers NIBRIV0000912523 (*H. dentata*), NIBRIV0000912521 (*O. inermis*), and NIBRIV0000912617 (*P. rathbuni*).

Next-generation sequencing was carried out using 350–450 bp paired-end libraries and Illumina Sequencing By synthesis. Initial quality checks were conducted using FastQC (v0.11.5; http://www.bioinformatics.babraham.ac.uk/projects/fastqc). Adapter trimming and removal of low-quality bases were performed with Trimmomatic (v0.36; Bolger et al. 2014). De novo assembly was undertaken in SPAdes (Bankevich et al. 2012; http://cab.spbu.ru/software/spades/), after which BLAST (Altschul et al. 1990; https://blast.ncbi.nlm.nih.gov/Blast.cgi) searches were used to identify mitochondrial contigs. Mitochondrial genome annotation, including detection of 13 protein-coding genes (PCGs), 22 tRNAs, and two rRNAs, was performed in MitoZ (Meng et al. 2019; https://github.com/linzhi2013/MitoZ). Then, each mitogenome was then visualized as a circular map using the web server Proksee (https://proksee.ca – Grant et al. 2023).

Open reading frames (ORFs) for the PCGs were verified using Geneious Prime v.2023.1.2 (Biomatters, Auckland, New Zealand) by comparison with previously reported anomuran sequences. The rrnL and rrnS genes were identified via MitoZ and cross-checked against other Anomura rRNA references. tRNA genes were predicted by MitoZ; their secondary structures were examined in ARWEN (Laslett and Canbäck 2008) and further refined using the structure editor in RNAstructure (Reuter and Mathews 2010). Then visualized using the program Forna (http://rna.tbi.univie.ac.at/forna- Kerpedjiev et al. 2015).

### Phylogenetic analyses based on mitochondrial protein-coding genes

For phylogenetic inference, 42 Anomura mitochondrial genomes (39 retrieved from GenBank and 3 newly sequenced herein) were analyzed, along with three brachyuran crabs *Portunus trituberculatus* (Portunoidea), *Dynomene pilumnoides* (Dromioidea), and *Homologenus malayensis* (Homoloidea) as outgroups (Table 1). Nucleotide and amino acid compositions for the 13 mitochondrial protein-coding genes were estimated in MEGA X, and AT/GC skew values were calculated following Perna and Kocher (1995). Codon usage was assessed via the Sequence Manipulation Suite v.2 (http://www.geneinfinity.org/sms/sms_codonusage.html). Multiple sequence alignments of the 13 PCGs were performed in Geneious Prime using Translation Align, and the concatenated dataset was partitioned by gene. The best-fit substitution model for each partition was identified with jModelTest 2.1.7 (Posada 2008) under the Bayesian Information Criterion (BIC). Phylogenies were reconstructed using two approaches: (1) Maximum Likelihood (ML) with 1,000 bootstrap replicates in RAxML v.8.2.9 (Stamatakis 2014), and (2) Bayesian Inference (BI) in MrBayes v.3.2.6 (Huelsenbeck and Ronquist 2001) via the CIPRES web portal (Miller et al. 2010).

**Table 1.** Complete mitochondrial genomes used for phylogenetic analysis in this study.

**Species**	**Infraorder**	**Superfamily**	**Family**	**Size (bp)**	**Accession no.**
Hapalogaster dentata*	Anomura	Lithodoidea	Lithodidae	16,607	OR523818
Oedignathus inermis*				16,584	OR523819
Pagurus rathbuni*		Paguroidea	Paguridae	16,466	OR523820
*Pagurus filholi*				15,674	LC222528
*Pagurus gracilipes*				16,051	LC222534
*Pagurus japonicus*				16,401	LC222532
*Pagurus lanuginosus*				14,632	LC222527
*Pagurus longicarpus*				15,630	NC003058
*Pagurus maculosus*				15,420	LC222526
*Pagurus minutus*				14,939	LC222533
*Pagurus nigrofascia*				15,423	MH756635
*Lithodes nintokuae*		Lithodoidea	Lithodidae	15,731	NC_024202
*Paralithodes brevipes*				16,303	AB735677
*Paralithodes camtschaticus*				16,720	JX944381
*Paralithodes platypus*				16,883	KY885248
*Birgus latro*		Paguroidea	Coenobitidae	16,411	KY352241
*Coenobita brevimanus*				16,393	MK310257
*Coenobita clypeatus*				16,469	ON203128
*Coenobita perlatus*				16,447	KY352234
*Coenobita rugosus*				16,427	KY352235
*Coenobita variabilis*				16,421	KY352236
*Clibanarius infraspinatus*			Diogenidae	16,504	NC_025776
*Dardanus arrosor*				16,592	MW147148
*Dardanus aspersus*				16,916	MW715812
*Pylocheles mortensenii*			Pylochelidae	15,093	KY352242
*Kiwa tyleri*		Chirostyloidea	Kiwaidae	16,865	KY423514
*Sternostylus investigatoris*			Sternostylidae	16,423	KY352237
*Sternostylus rogeri*				16,504	KY352238
*Aegla longirostri*		Aegloidea	Aeglidae	15,387	MF457407
*Lomis hirta*		Lomisoidea	Lomisidae	17,239	KY352239
*Allogalathea elegans*		Galatheoidea	Galatheidae	16,253	ON968875
*Curtonida isos*				17,910	MF457406
*Grimothea gregaria*				16,326	KU521508
*Munidopsis lauensis*				17,483	MH717895
*Munidopsis verrilli*				17,636	MH717896
*Shinkaia crosnieri*				15,182	EU420129
*Petrolisthes haswelli*				15,348	LN624374
*Pisidia serratifrons*				15,344	OM461359
*Neopetrolisthes maculatus*				15,324	KC107816
*Stemonopa insignis*		Hippoidea	Albuneidae	15,596	KY352240
*Blepharipoda liberata*			Blepharipodidae	15,766	OK514627
*Emerita talpoida*			Hippidae	15,810	ON164669
*Portunus trituberculatus*	Brachyura (Outgroup)	Portunoidea	Portunidae	16,017	MW232435
*Dynomene pilumnoides*		Dromioidea	Dynomenidae	16,475	KT182070
*Homologenus malayensis*		Homoloidea	Homolidae	15,793	NC_026080

For BI, two independent Markov chain Monte Carlo (MCMC) runs of 10^6^ generations (six chains each) were performed, sampling every 100 generations. The initial 2 × 10^3^ trees were discarded as burn-in after stationarity was confirmed, and Bayesian posterior probabilities (BPP) were calculated from the remaining trees.

### Selection analysis of mitochondrial PCGs

Selective pressure analyses of protein-coding genes (PCGs) began with codon-based alignments using MUSCLE (Edgar 2004) in MEGA 11 (Tamura et al. 2021). For each gene, the respective values of K_a_ (nonsynonymous substitutions per nonsynonymous site; K_a_ = d_N_ = S_A_/L_A_), K_s_ (synonymous substitutions per synonymous site; K_s_ = d_S_ = S_S_/L_S_), and *ω* (K_a_/K_s_) were estimated using K_a_K_s__calculator 2.0 (Wang et al. 2010) to identify regions potentially under positive selection. A K_a_K_s_ ratio was interpreted as follows: K_a_K_s_ = 1 where PCGs experience neutral selection, K_a_K_s_ > 1 where PCGs experience positive (diversifying) selection, or K_a_K_s_ < 1 where PCGs experience negative (purifying) selection. The *γ-MYN* model was employed to account for variation in mutation rates across sequence sites (Wang et al. 2009).

To explore potential associations with depth-dependent adaptation, we performed pairwise comparisons among species inhabiting similar depth ranges within the same family, focusing on three newly reported species (*Hapalogaster dentata*, *Oedignathus inermis*, and *Pagurus rathbuni*) (Table 2). For *P. rathbuni*, which belongs to the family Paguridae, the following three comparisons were conducted: *P. gracilipes* and *P. rathbuni* (shallow-water <60 m vs. deep-water 60–590 m), *P. lanuginosus* and *P. maculosus* (both intertidal species, <5 m), *Pagurus gracilipes* and *Pagurus filholi* (shallow-water <60 m vs. intertidal <2 m). For *H. dentata* and *O.* *inermis*, members of the family Lithodidae, the following two comparisons were made: (1) *H. dentata* and *O. inermis* (both inhabiting shallow depths <10 m and <45 m), (2) *Paralithodes camtschaticus* and *Paralithodes platypus* (shallow-to-moderate depths: 10–180 m vs. 10–280 m). To identify positively selected sites along each examined sequence, K_a_, K_s_, and *ω* values were further calculated using a sliding window approach (window length = 52, step size = 12).

**Table 2.** Pairwise comparisons among closely related species within Paguridae and Lithodidae, grouped by depth range, used to evaluate depth-dependent adaptation, including three newly reported species. Selective pressure analysis of all PCGs was conducted using the KaKs_Calculator program.

***Pagurus gracilipes*** **&** ***P. rathbuni***					
**PCG No.**	**PCG**	**Ka**	**Ks**	**Ka/Ks**	***P*-Value(Fisher)**
1	COX1	0.00331028	4.24514	0.00077978	1.01E-193
2	COX2	0.018025	3.6157	0.0049852	9.42E-78
3	NAD3	0.00352287	1.77398	0.00198586	1.17E-30
4	NAD2	0.0331438	1.50058	0.0220873	5.00E-83
5	ATP8	0.0627215	1.926	0.0325656	4.51E-07
6	ATP6	1.99878	2.35465	0.848867	0.543361
7	COX3	0.149899	3.31035	0.045282	4.83E-40
8	NAD5	0.0266794	1.64894	0.0161798	1.99E-120
9	NAD4	0.0259642	1.09275	0.0237603	2.36E-81
10	NAD4L	0.0126535	1.08347	0.0116787	6.22E-25
11	NAD6	0.0482835	1.43984	0.0335339	5.84E-29
12	CYTB	0.00767532	2.4861	0.0030873	6.95E-128
13	NAD1	0.00528339	2.04807	0.00257969	1.64E-106
***Pagurus lanuginosus* & *P. maculosus***					
**PCG No.**	**PCG**	**Ka**	**Ks**	**Ka/Ks**	***P*-Value(Fisher)**
1	COX1	0.00852892	1.13071	0.00754295	4.13E-62
2	COX2	1.11E-15	1.09625	1.01275E-15	0
3	NAD3	0.00356031	0.946543	0.00376139	1.75E-15
4	NAD2	0.0123047	0.491812	0.0250191	1.35E-28
5	ATP8	0.0122317	0.829653	0.0147431	2.64E-05
6	ATP6	0.00531749	0.671454	0.00791937	3.54E-26
7	COX3	0.0282069	0.626544	0.0450198	3.85E-21
8	NAD5	0.0120074	0.91321	0.0131486	6.41E-64
9	NAD4	0.0277932	1.00686	0.0276038	6.06E-37
10	NAD4L	0.0296945	0.578929	0.0512921	4.34E-07
11	NAD6	0.018086	0.573016	0.0315628	1.29E-14
12	CYTB	0.00883624	0.519777	0.017	1.58E-40
13	NAD1	0.0257692	1.55037	0.0166213	3.03E-32
***Pagurus gracilipes* & *P. filholi***					
**PCG No.**	**PCG**	**Ka**	**Ks**	**Ka/Ks**	***P*-Value(Fisher)**
1	COX1	0.000787776	1.18356	0.000665599	3.20E-98
2	COX2	0.0035202	1.08314	0.00324999	1.05E-42
3	NAD3	0.0105117	0.513658	0.0204645	3.15E-14
4	NAD2	0.0192254	0.753799	0.0255047	2.15E-39
5	ATP8	0.00667893	0.374335	0.0178421	2.03E-05
6	ATP6	1.11E-15	0.444066	2.50E-15	0
7	COX3	0.0849805	0.953383	0.0891357	2.56E-13
8	NAD5	0.0121031	0.548115	0.0220813	4.38E-63
9	NAD4	0.0105959	0.44791	0.0236563	3.06E-52
10	NAD4L	0	1.33834	0	0
11	NAD6	0.0199635	0.431598	0.0462548	3.22E-19
12	CYTOB	0.00539174	0.59463	0.00906739	3.53E-50
13	NAD1	0.00506445	0.580698	0.00872132	1.22E-38
***Hapalogaster dentata* & *Oedignathus inermis***					
**PCG No.**	**PCG**	**Ka**	**Ks**	**Ka/Ks**	***P*-Value(Fisher)**
1	COX1	0.00490383	1.70743	0.00287205	1.49E-167
2	NAD1	0.00408507	1.17471	0.00347752	3.60E-89
3	CYTOB	0.0184755	3.89935	0.00473811	1.06E-127
4	NAD6	0.132764	1.47755	0.0898539	2.36E-24
5	NAD4L	0.00419715	2.78738	0.00150577	7.62E-29
6	NAD4	0.0306301	1.29289	0.0236911	3.07E-89
7	NAD5	0.0359908	2.38012	0.0151214	6.82E-119
8	COX3	0.0185626	3.66343	0.00506701	6.28E-90
9	ATP6	0.0132453	3.68203	0.00359727	1.72E-81
10	ATP8	0.0840003	2.05081	0.0409595	5.97E-09
11	NAD2	0.0728642	1.15628	0.063016	6.70E-51
12	NAD3	0.0257714	1.25041	0.0206104	2.95E-23
13	COX2	0.0113276	1.38817	0.00816012	1.09E-55
***Paralithodes camtschaticus* & *P. platypus***					
**PCG No.**	**PCG**	**Ka**	**Ks**	**Ka/Ks**	***P*-Value(Fisher)**
1	COX1	0.00160315	1.2401	0.00129276	2.63E-85
2	COX2	0.00521527	1.25128	0.00416795	3.63E-40
3	NAD3	0.00338361	1.18088	0.00286533	4.77E-23
4	NAD2	0.0112643	1.00482	0.0112102	1.46E-64
5	ATP8	0.00758026	0.165077	0.0459196	0.00825718
6	ATP6	0.00179824	0.735927	0.00244351	9.58E-39
7	COX3	0.00912788	1.14413	0.007978	3.00E-48
8	NAD5	0.0173105	1.4019	0.0123479	7.90E-92
9	NAD4	0.00443452	1.66078	0.00267013	2.35E-85
10	NAD4L	1.11E-15	0.689819	1.61E-15	0
11	NAD6	0.0191781	1.1435	0.0167714	1.64E-24
12	CYTOB	0.00333551	0.571791	0.00583345	1.21E-59
13	NAD1	0.00385775	0.826969	0.00466494	1.05E-52

### Divergence time estimation and time tree construction

Divergence times within Anomura were inferred using all 13 PCGs in BEAST v2.6.3 (Bouckaert et al. 2019). A calibrated Yule model was selected for the tree prior, and optimal evolutionary models were assessed via jModelTest 2.1.7. Fossil constraints based on 18 reference points (Table 3) were applied to calibrate nodal ages. Three independent MCMC runs of 10^7^ generations each were conducted, sampling every 200 generations. Convergence was evaluated in Tracer v1.7.1 (Rambaut et al. 2018), discarding the initial 10% of samples as burn-in. The log and tree files from each run were combined in LogCombiner and TreeAnnotator (part of the BEAST package), and the maximum clade credibility (MCC) tree was visualized in FigTree v1.4.4 (Rambaut 2018).

**Table 3.** Fossil calibrations used in BEAST divergence time analyses in this study modified from Bracken-Grissom et al. 2013.

**Taxonomy**	**Species**	**Geological Age (MYA)**	
Infraorder Anomura	*Platykotta akaina* Chablais, Feldmann and Schweitzer, 2011	Late Triassic (Norian/Rhaetian) 201.6–228	
Superfamily Aegloidea			
Family Aeglidae	*Protaegla miniscula* Feldmann, Vega, Applegate, and Bishop, 1998	Early Cretaceous (Albian) 99.6–112	
Superfamily Chirostyloidea	*Pristinaspina gelasina* Schweitzer and Feldmann, 2001	Late Cretaceous 65.5–99.6	
Superfamily Galatheoidea			
Family Galatheidae	*Galatheites zitteli* (Moericke, 1889)	Late Jurassic (Tithonian) 145.5–151	
Family Munididae	*Juracrista perculta* Robins, Feldmann, and Schweitzer, 2012	Late Jurassic (Tithonian) 145.5–151	
Family Munidopsidae	*Gastrosacus wetzleri* Von Meyer, 1851	Late Jurassic (Oxfordian-Tithonian) 161–145	
Genus *Munidopsis*	*Munidopsis foersteri* Feldmann et al., 1993	Late Cretaceous (Campanian) 70.6–83.5	
Genus *Shinkaia*	*Shinkaia katapsyxis* Schweitzer and Feldmann, 2008	Eocene 33.9–55.8	
Family Porcellanidae	*Jurellana tithonia* Schweitzer and Feldmann, 2010	Late Jurassic (Tithonian) 145.5–151	
Genus *Petrolisthes*	*Petrolisthes bittneri* De Angeli and Garassino, 2002	Oligocene 23.7–36.6	
Genus *Pisidia*	*Pisidia dorsosinuata* De Angeli and Garassino, 2002	Eocene 36.6–57.8	
Superfamily Hippoidea			
Family Blepharipodidae	*Lophomastix antiqua* Schweitzer and Boyko, 2000	Eocene 33.9–55.8	
Superfamily Lithodoidea			
Family Lithodidae	*Paralomis debodeorum* Feldmann, 1998	Miocene 5.3–23	
Superfamily Paguroidea	*Palaeopagurus deslongchampsi* Van Straelen, 1925	Early Jurassic (Pliensbachian) 190–183	
Family Coenobitidae	*Birgus latro* Linnaeus, 1767	Pliocene 2.6–5.3	
Family Diogenidae	*Annuntidiogenes ruizdegaonai* Fraaije et al., 2008	Early Cretaceous (Albian) 99.6–112	
Family Paguridae	*Pagurus malloryi* Schweitzer and Feldmann 2001	Oligocene 23.7–36.6	
Family Pylochelidae	*Jurapylocheles malutka, Ammopylocheles mclaughlinae* Van Bakel et al. 2008	Late Jurassic (Kimmeridgian) 151–156	

### Analysis of structure of control region

Tandem repeats within the putative D-loop/control region were identified using Tandem Repeat Finder v.4.09 (Benson 1999; http://tandem.bu.edu/trf/trf.html) under default settings. DNA motifs were detected with MEME (Bailey et al. 2009), and inverted repeats were searched using EMBOSS ‘einverted’ (Rice et al. 2000; http://www.bioinformatics.nl/cgi-bin/emboss/einverted) with default parameters. The Microsatellite Repeats Finder (Bikandi et al. 2004; http://insilico.ehu.es/mini_tools/microsatellites) web server was used to locate simple sequence repeats (SSRs). Secondary structures in this region were predicted via the RNAstructure web server (Reuter and Mathews 2010; http://rna.urmc.rochester.edu/RNAstructureWeb/) at 27 °C under default settings, focusing on the presence of stem-loops.

## Results

### General features of the three newly sequenced Anomuran mitochondrial genomes

**Figure 1.**
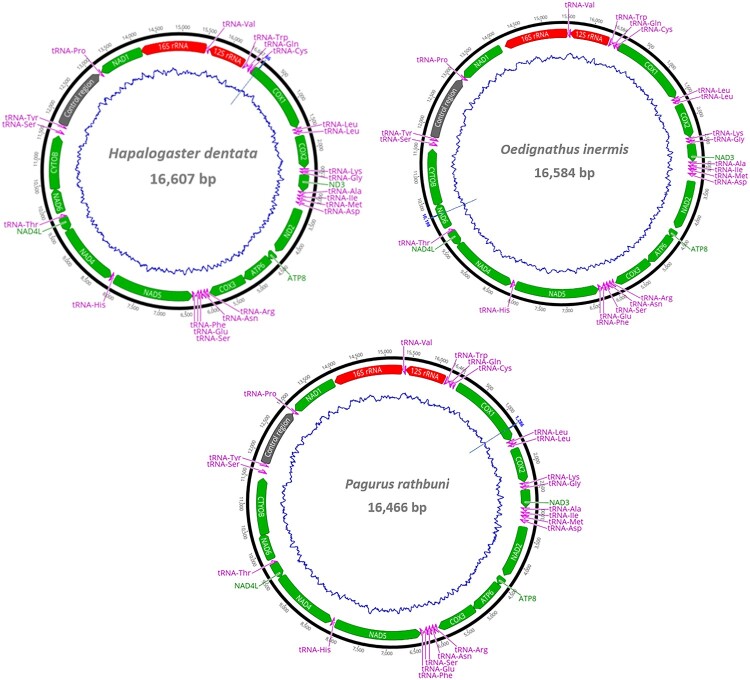
Circular mitochondrial genome maps of three anomurans. Arrows indicate gene transcription direction. tRNAs are denoted by single-letter amino acid abbreviations. Two leucine and two serine tRNA genes are labeled by their anticodon sequences: L1 (trnL1-tag), L2 (trnL2-taa), S1 (trnS1-tct), and S2 (trnS2-tga). The blue line graph within the genome maps represents GC content.

The mitochondrial genomes of *Hapalogaster dentata* (16,607 bp), *Oedignathus inermis* (16,600 bp), and *Pagurus rathbuni* (16,466 bp) each encoded for 13 protein-coding genes (PCGs), 22 transfer RNA, and two ribosomal RNA (Figure 1, Figure 3). Additionally, more than half of the 22 *tRNA* genes were predicted to adopt atypical cloverleaf secondary structures in each species (*H. dentata*: 12; *O. inermis*: 16; *P. rathbuni*: 16) (Supplementary Figures S1).

Base compositions also deviated from established crustacean norms (Hassanin 2006). In *H. dentata*, the heavy strand exhibited A = 31.7%, T = 41.8%, C = 9.8%, and G = 16.7% (G + C = 26.5%), whereas the light strand had A = 38.6%, T = 30.7%, C = 14.1%, and G = 16.6%. Consequently, the AT skew was –0.137 and the GC skew was 0.081. Similar patterns were observed in *O. inermis* (heavy strand: A = 31.2%, T = 42.2%, C = 9.6%, G = 17.0%; light strand: A = 37.7%, T = 31.2%, C = 13.3%, G = 17.8%), yielding AT and GC skew values of –0.149 and 0.145, respectively. In contrast, *P. rathbuni* displayed slightly lower A + T content (heavy strand: A = 29.2%, T = 38.5%, C = 17.0%, G = 15.3%; light strand: A = 39.9%, T = 33.1%, C = 16.0%, G = 11.0%), with AT and GC skews of –0.137 and –0.210.

### Phylogenetic relationships among Anomura

**Figure 2.**
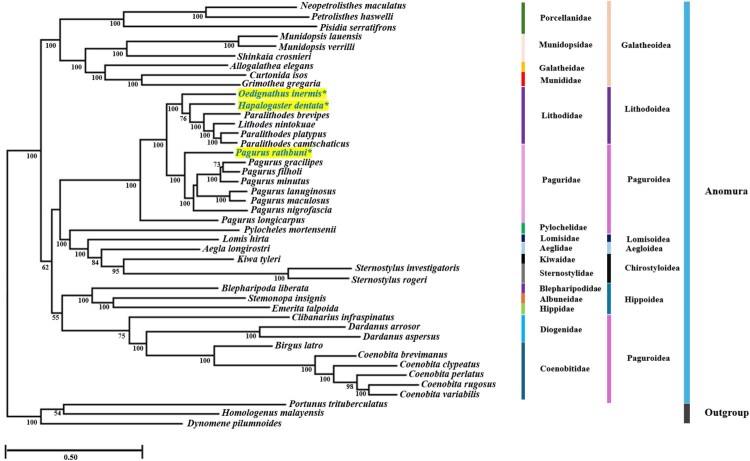
Mitochondrial genome phylogeny of 42 anomurans based on 13 PCGs using maximum likelihood and Bayesian methods. Maximum likelihood bootstrap (BP)/Bayesian posterior probability (BPP) values are shown near branches. Taxa with bold blue letters and asterisk (*) indicate newly determined mitochondrial genome sequences in this study.

The mitochondrial DNA (mtDNA)-based phylogenetic tree robustly supports the monophyly of Anomura (Figure 2). The position of the superfamily Hippoidea at the base of the Anomura clade indicates that it represents the earliest diverging lineage among the examined anomuran superfamilies. All three newly sequenced species (marked with asterisks) were placed within their families with high support. Among the superfamilies represented by three or more species, i.e. Lithodoidea, Chirostyloidea, Galatheoidea, and Hippoidea, all formed well-supported monophyletic clades. In contrast, Paguroidea was recovered as polyphyletic, forming three distinct clades: one composed of Coenobitidae and Diogenidae, another consisting solely of Paguridae, and the third formed exclusively by Pylochelidae. Notably, *Pagurus longicarpus* did not cluster with other *Pagurus* species, rendering the genus paraphyletic. Furthermore, Diogenidae was also paraphyletic, as it included members of Coenobitidae. At the genus level, non-monophyly was observed in several cases; for example, *Paralithodes* was recovered as paraphyletic.

### Comparative analysis of mitochondrial gene arrangements

**Figure 3.**
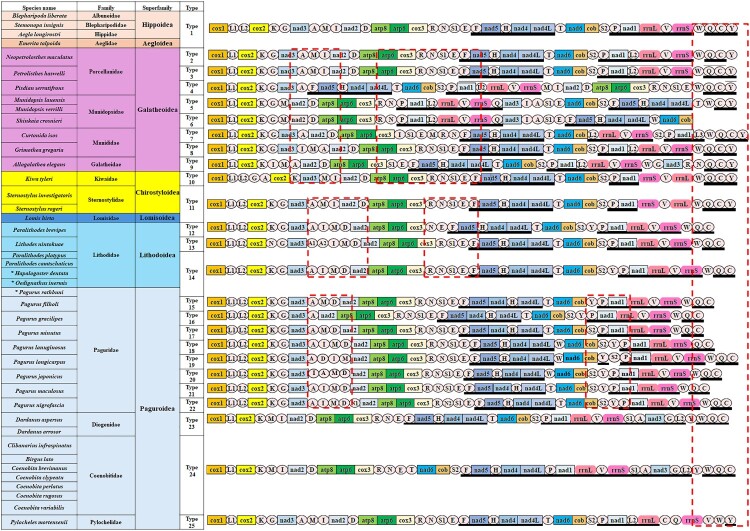
Mitochondrial gene order patterns of 42 anomurans combined with a phylogenetic tree (detailed phylogenetic relationships are shown in Figure 2). Gene and genome sizes are not to scale. tRNAs are denoted by single-letter amino acid abbreviations. The black bar below the genes indicates inverted genes. Taxa with blue letters and asterisk (*) indicate newly determined mitochondrial genome sequences in this study. Regions highlighted by red dashed lines correspond to rearrangements in gene order.

Within Anomura, a total of 25 distinct mitochondrial gene arrangement types were identified. Among these, 12 were observed in the superfamily Paguroidea, including nine within the family Paguridae, while the remaining three types were specific to the families Diogenidae, Coenobitidae, and Pylochelidae, each exhibiting a single unique gene arrangement (Figure 3).

The mitochondrial gene orders of the three newly sequenced species were compared primarily with those of confamilial taxa, and more broadly with other members of Anomura. Each synteny showed that the arrangement of protein-coding genes (PCGs) was consistent with that of previously reported species within the respective families. However, several *tRNA* genes exhibited translocations. Specifically, *H. dentata* and *O. inermis*, both belonging to Lithodidae, showed differences in the arrangement of (*tRNA*-*A*, *tRNA*-*M*, *tRNA*-*I*, *tRNA*-*D*) and (*tRNA*-*R*, *tRNA*-*N*, *tRNA*-*E*, *tRNA*-*F*). Previous studies (Tan et al. 2018, 2019; Wang et al. 2019, 2023; Veldsman et al. 2020) have also reported duplications or positional shifts of specific *tRNA* genes in lithodid species such as *Paralithodes brevipes* and *Lithodes nintokuae*. Within the family Paguridae, compared with other *Pagurus* species, *P. rathbuni* was found to exhibit tRNA gene rearrangements in the (*tRNA*-*A*, *tRNA*-*M*, *tRNA*-*I*, *tRNA*-*D*) and (*tRNA*-*Y*, *tRNA*-*S2*) regions (Figure 3). These findings are consistent with *tRNA* translocation patterns previously reported in *P. japonicus* and *P. longicarpus* based on mitochondrial genome analyses of other pagurid species (Zhang et al. 2021). Collectively, it is noteworthy that these three species from different families share the same mitochondrial gene arrangement (Type 14). This observation is consistent with the phylogenetic framework proposed by Bracken-Grissom et al. (2013), which demonstrated that Lithodidae is the closest family of Paguridae and supported the ‘hermit-to-king’ hypothesis. Moreover, the shared arrangement may provide valuable insights into reconstructing the ancestral state of the common ancestor of these two families. Additionally, species within superfamilies Hippoidea and Aegloidea were found to share a single conserved gene arrangement, whereas superfamily Galatheoidea exhibited considerable variability, showing eight distinct gene arrangement types across both family and genus levels. Therefore, although certain degrees of conservation are present at specific phylogenetic levels, a consistent ground pattern for Anomura mitochondrial gene arrangements has yet to be established.

### Mitochondrial time tree of Anomura

**Figure 4.**
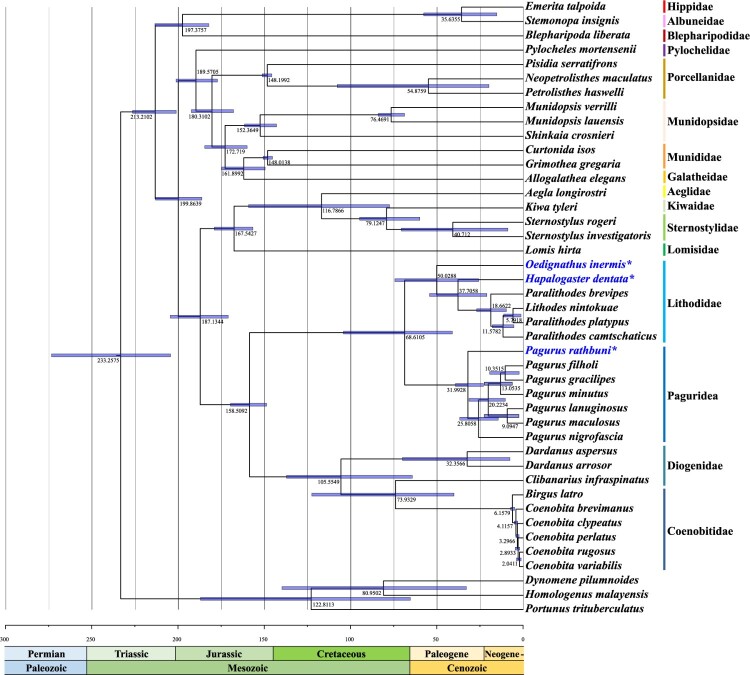
Time tree with mean age estimates (with 95% HPD intervals) based on 13 PCGs of 40 anomuran mitochondrial genomes. Taxa with blue letters and asterisk (*) indicate newly determined mitochondrial genome sequences in this study.

We estimated divergence times within Anomura using 13 protein-coding genes (PCGs) under a fossil-calibrated molecular clock framework (Figure 4). The split between infraorders Anomura and Brachyura was dated to ∼233 million years ago (MYA) during the Upper Triassic (Norian). Superfamily Hippoidea was the earliest lineage to diverge (∼213 MYA, Rhaetian), followed by the superfamily Galatheoidea–family Pylochelidae clade (∼200 MYA, Sinemurian), and subsequently the superfamilies Aegloidea–Lomisoidea–Chirostyloidea (∼187 MYA, Toarcian). The final major divergence involved the families Diogenidae, Coenobitidae, Paguridae, and Lithodidae, occurring around 159 MYA (Kimmeridgian). Our analysis indicates that two Hapalogastrinae species (*H. dentata* and *O. inermis*) diverged earlier (38–50 MYA, Eocene) than other lithodid species, whereas *P. rathbuni* diverged approximately 32 MYA (Oligocene). These findings suggest that major evolutionary transitions in these lineages coincided with the climatic upheavals occurring around the Eocene–Oligocene boundary.

### Comparison of the characteristics of the control region

We compared the length, location, AT content, presence of repeats, and free energy values of control region across species belonging to 17 families within six superfamilies of the infraorder Anomura, along with their habitat depth and climatic conditions (Table 4). For broader ecological comparison, we additionally included species from three families within three superfamilies (Portunoidea, Dromioidea, and Homoloidea) of the infraorder Brachyura, as well as representative species from the infraorders Achelata (Palinuridae), Caridea (Alpheoidea), terrestrial Isopoda, and Stomatopoda. Excluding tandem repeats, the remaining features are as follows: The length was highly compact, ranging from cases where it was absent or extremely short, such as 63 bp in *P. filholi* (Paguridae), to as long as 1557 bp in *Curtonida isos* (Munididae). The control region was generally located between *rrnS* and *tRNAS*, but its position varied depending on the diversity of gene order. The AT content ranged from a minimum of 61.50% in *Birgus latro* (Coenobitidae) to a maximum of 90.50% in *Kiwa tyleri* (Kiwaidae). Lastly, the free energy values suggested consistent patterns when considered broadly at the family level: In Diogenidae and Paguridae, Δ*G* tended to decrease (i.e. become more negative) with increasing depth. Conversely, species in Lithodidae exhibited increasing Δ*G* values at greater depths. Although Munidopsidae species were found in the deepest habitats, including hydrothermal vents, they displayed relatively high Δ*G* values (i.e. low absolute magnitude). In contrast, no consistent depth-related trends in Δ*G* were observed in Porcellanidae and Hippoidea.

**Table 4.** Comparative characteristics of the mitochondrial control region across species representing 17 families within six superfamilies of Anomura, including length, location, AT content, repeat presence, and free energy values, together with habitat depth and climatic conditions.

**Order/Infraorder**	**Superfamily**	**Family/subfamily**	**Species [Reference]**	**Length (bp)**	**Location**	**%AT**	**Repeat (Copy number × Period Size)**	***ΔG* value (kcal/mol)**	**Habitat depth**	**Habitat climate**
**Decapoda; Anomura**	**Paguroidea**	Lithodidae; Hapalogastrinae	* *Hapalogaster dentata*	1,313	between *trnP* and *trnY*	71.60%	3.6 × 47 bp	−396.1 to −395.1	0–10 m	Temperate
			* *Oedignathus inermis*	1,300		74.10%	3.5 × 47 bp	−416.7 to −415.4	0–45 m	
		Lithodidae; Lithodinae	*Paralithodes platypus* [KY885248]	1,422		75.10%	11.9 × 10 bp, 7.4 × 20 bp, 2.1×48 bp, 3.4×48 bp	−267.9 to −267.1	10–180 m	Cold
			*Paralithodes camtschaticus* [JX944381]	1,428		77.90%	6.6 × 10 bp, 11.2 × 12 bp, 3.4 × 48 bp	−192.6 to −192.1	10–200 m	
			*Paralithodes brevipes* [AB735677]	979		72.20%	3.5 × 48 bp	−130.2 to −129.2	10–280 m	
			*Lithodes nintokuae* [NC024202]	275	between *trnS2* and *trnP*	86.00%	3.1 × 51 bp, 2.1×76 bp, 6.2×25 bp	−19.7 to −17.8	450–1,070 m	
		Coenobitidae	*Birgus latro* [KY352241]	1,381	between *rrnS* and *trnS1*	61.50%	No Repeat	−297.1 to −295.8	Terrestrial	Tropic and subtropical
			*Coenobita brevimanus* [MK310257]	1,377	between *rrnS* and *trnS2*	62.10%	No Repeat	−321.5 to −320.6		
			*Coenobita clypeatus* [ON203128]	1,390		65.00%	No Repeat	−244.8 to −243.8		
			*Coenobita perlatus* [KY352234]	1,356	between *rrnS* and *trnS1*	68.10%	2.0 × 24 bp	−271.7 to −270.7		
			*Coenobita rugosus* [KY352235]	1,366		65.60%	2.2 × 18 bp	−264.9 to −263.8		
			*Coenobita variabilis* [KY352236]	1,371		65.90%	No Repeat	−254.8 to −254.0		
		Diogenidae	*Clibanarius infraspinatus* [NC025776]	1,500		69.40%	No Repeat	−179.1 to −178.3	Terrestrial (0–1 m)	
			*Dardanus aspersus* [MW715812]	1,461	between *rrnS* and *trnS2*	63.90%	No Repeat	−784.3 to −781.8	20–60m	
			*Dardanus arrosor* [MW147148]	1,496		62.40%	No Repeat	−796.4 to −793.2	5–750m	
		Paguridae	* *Pagurus rathbuni*	1,124	between *trnP* and *trnY*	70.00%	3.6 × 47 bp	−322.5 to −321.0	60–590m	Cold
			*Pagurus gracilipes* [LC222534]	1,120		73.00%	2.0 × 21 bp	−180.5 to −179.5	0–60m	Temperate
			*Pagurus nigrofascia* [MH756635]	106		75.50%	No Repeat	−6.6 to −6.0	0–5m	
			*Pagurus lanuginosus* [LC222527]	122		86.00%	4.2 × 10 bp	−5.0 to 6.6		
			*Pagurus maculosus* [LC222526]	150		80.70%	No Repeat	−11.4 to 8.3		
			*Pagurus japonicus* [LC222532]	484		78.90%	No Repeat	−49.8 to −48.2	5–300m	
			*Pagurus longicarpus* [AF150756]	530		65.30%	3.2×45 bp	−80.6 to −79.3	0–200m	Temperate and subtropical
			*Pagurus minutus* [LC222533]	Too compact					0–2m	Temperate
			*Pagurus filholi* [LC222528]	63	between *rrnL* and *trnV*	76.20%	No Repeat	−2.9 to 4		
		Pylochelidae	*Pylocheles mortensenii* [KY352242]	Too compact					100–627m	Tropic
	**Hippoidea**	Albuneidae	*Stemonopa insignis* [KY352240]	605	between *trnS2* and *trnP*	67.60%	No Repeat	−63.6 to −62.3	3–45m	
		Hippidae	*Emerita talpoida* [ON164669]	553	between *trnS2* and *trnP*	75.40%	No Repeat	−62.4 to −60.4	0–2m	
		Blepharipodidae	*Blepharipoda liberata* [OK514627]	611	between *trnS2* and *trnP*	81.50%	No Repeat	−180.5 to −179.5	2–10m	Temperate
	**Galatheoidea**	Porcellanidae	*Petrolisthes haswelli* [LN624374]	603	between *rrnS* and *trnW*	75.50%	No Repeat	−70.8 to −69.6	0–5m	Tropical
			*Neopetrolisthes maculatus* [KC107816]	547		75.20%	No Repeat	−67.8 to −65.6	0–30m	
			*Pisidia serratifrons* [OM461359]	371	between *rrnS* and *trnM*	83.00%	53 × 2 bp	−56.6 to −54.9	2–15m	Temperate
		Galatheidae	*Allogalathea elegans* [ON968875]	1,037	between *trnE* and *trnF*	75.10%	No Repeat	−44.1 to −43.2	0–146m	
		Munididae	*Curtonida isos* [MF457406]	1,557	between *rrnS* and *trnS2*	65.80%	2.4 × 11 bp, 2.1 × 292 bp	−77.3 to −76.7	462–2,756m	
			*Grimothea gregaria* [KU521508]	634	between *rrnS* and *trnW*	80.70%	No Repeat	−79.4 to −77.5	34–200m	Temperate and subtropical
		Munidopsidae	*Shinkaia crosnieri* [EU420129]	327	between *rrnS* and *trnQ*	83.50%	No Repeat	−23.5 to −21.9	1,200–1,500m	Deep-sea hydrotherm al vents in tropical and subtropical regions
			*Munidopsis lauensis* [MH717895]	650		73.40%	1.9 × 22 bp	−38.7 to −37.3	1,649–2,000m	
							2.7 × 13 bp			
							2.3 × 26 bp			
							5.5 × 6 bp			
			*Munidopsis verrilli* [MH717896]	647		75.30%	3.0×11 bp	−58.3 to −57.2	1,575–4,169m	
							1.9×15 bp			
							3.9×12 bp			
							2.3×19 bp			
	**Chirostyloidea**	Kiwaidae	*Kiwa tyleri* [KY423514]	1,525	between *rrnL* and *trnW*	90.50%	(1.9–19.5) × (2–36) bp: 21 times	−198.7 to −197.8	1,000–2,400m	
		Chirostylidae	*Sternostylus investigatoris* [KY352237]	1,325	between *rrnL* and *trnW*	65.70%	2.1 × 92 bp	−175.7 to −175.0	299–957 m	Tropical
			*Sternostylus rogeri* [KY352238]	1,395		71.40%	No Repeat	−152.5 to −151.9	1,364 m	
	**Lomisoidea**	Lomisidae	*Lomis hirta* [KY352239]	2,036		73.40%	2.6 × 119 bp	−133.5 to −132.8	0–5m	Temperate
	**Aegloidea**	Aeglidae	*Aegla aff. longirostri* [MF457407]	428	between *nad1* and *rrnL*	65.30%	No Repeat	−46.1 to −46.2	0–1m	Fresh water
**Decapoda; Brachyura**	**Portunoidea**	Portunidae	*Portunus trituberculatus* [MW232435]	1,532	between *trnY* and *trnL1*	64.50%	No Repeat	−167.9 to −166.9	0–50m	Temperate and subtropical
	**Dromioidea**	Dynomenidae	*Dynomene pilumnoides* [KT182070]	652	between *trnL2* and *trnL*	80.40%	No Repeat	−74.6 to −73.2	10–100m	
	**Homoloidea**	Homolidae	*Homologenus malayensis* [NC026080]	883	between *rrnS* and *trnS2*	75.60%	9.9 × 42 bp	−134.1 to −133.6	50–600m	Tropic and subtropical
**Decapoda; Achelata**		Palinuridae	*Panulirus argus* [Baeza 2018]	801	between *rrnS* and *trnI*	69.60%	No Repeat	−99.20 to −94.52	0–90 m	Tropic
**Decapoda; Caridea**	**Alpheoidea**	Alpheidae	*Synalpheus microneptunus* [Chak et al. 2020]	834		79.50%	No Repeat	−104 (lowest)	0–23 m	
**Isopoda**		Armadillidae	*Cubaris murina* [Hwang et al. 2024]	370	between *trnS1* and *trnL1*	59.70%	No Repeat	−103.7 to −101.0	Terrestrial	Tropic and subtropical
**Stomatopoda**	**Parasquilloidea**	Parasquillidae	*Faughnia haani* [Hwang et al. 2021]	1,162	between *rrnS* and *trnI*	74.00%	3.1 × 137 bp	−138.9 to −137.8	72–230 m	

**Figure 5.**
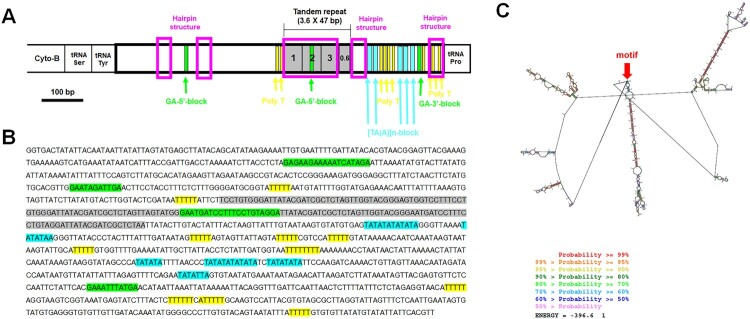
Characteristics of the control region of *Hapalogaster dentata*. The putative conserved elements are highlighted: A-B, One fragment (47 bp long) found to repeat 3.6 times between 1 and 169 bp is shown in gray. Three GA-blocks (two GA-5′-block and one GA-3′-block) are marked by green. The poly T stretch is marked by yellow, the hairpin structures are marked by the pink. The [TA(A)]n-blocks which is known that it can appear multiple times are marked by the fluorescent blue. C. The secondary structure of this region at its lowest energy value. Red arrows denote the positions of motifs.

**Figure 6.**
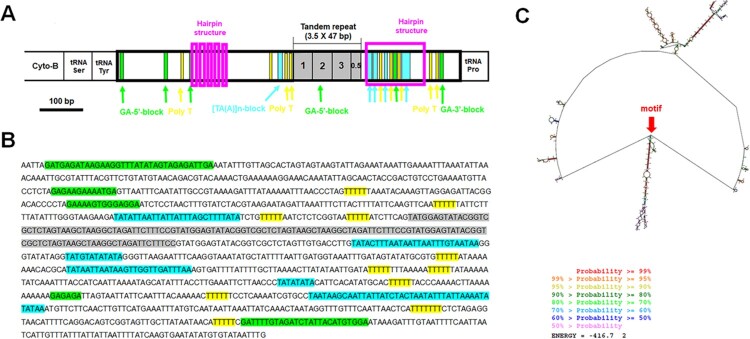
Characteristics of the control region of *Oedignathus inermis*. The putative conserved elements are highlighted: A-B, One fragment (47 bp long) found to repeat 3.5 times between 1 bp and 164 bp is shown in gray. Four GA-blocks (three GA-5′-block and one GA-3′-block) are marked by green. The poly T stretch is marked by yellow, the hairpin structures are marked by the pink. The [TA(A)]n-blocks which is known that it can appear multiple times are marked by the fluorescent blue. C. The secondary structure of this region at its lowest energy value. Red arrows denote the positions of motifs.

**Figure 7.**
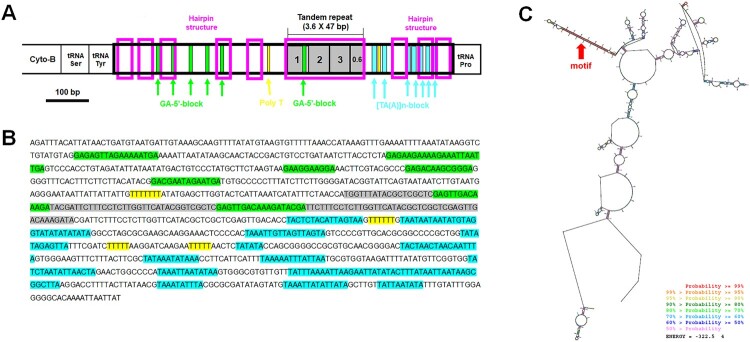
Characteristics of the control region of *Pagurus rathbuni.* The putative conserved elements are highlighted: A-B, One fragment (47 bp long) found to repeat 3.6 times between 1 and 169 bp is shown in gray. Six GA-blocks (five GA-5′-block and one GA-3′-block) are marked by green. The poly T stretch is marked by yellow, the hairpin structures are marked by the pink. The [TA(A)]n-blocks which is known that it can appear multiple times are marked by the fluorescent blue. C. The secondary structure of this region at its lowest energy value. Red arrows denote the positions of motifs.

Visual examination of the control region and the use of the web server Tandem Repeat Finder enabled the detection of a 47 bp fragment that was repeated 3.5–3.6 times (Figures 5–7). In all three species (*H. dentata*, *O. inermis*, and *P. rathbuni*) the control region is located between *tRNA-P* and *tRNA-Y*, and RNAstructure consistently predicted 20 possible secondary structures. Detailed information for each species is as follows: In *H. dentata*, the control region is a 1,313-bp intergenic segment (A = 32.6%, T = 39.0%, C = 12.0%, G = 16.4%) that contains a 47-bp repeat motif repeated approximately 3.6 times (positions 417–584 bp; Figures 1 and 5). Several GA blocks, poly-T stretches, and [TA(A)]_n_ blocks were also detected, with predicted *ΔG* values ranging from –396.1 to –395.1 kcal/mol (Supplementary Figure S2). Similarly, in *O. inermis*, the 1,300-bp control region (A = 35.3%, T = 38.8%, C = 11.5%, G = 14.4%) harbors a 47-bp repeat motif repeated ∼3.5 times (positions 463–628 bp; Figure 6, Table 4), along with GA blocks, multiple poly-T stretches, and hairpin structures. The predicted *ΔG* values range from –322.5 to –321.0 kcal/mol (Supplementary Figure S3). In *P. rathbuni*, the control region spans 1,124 bp (A = 34.8%, T = 31.2%, C = 18.2%, G = 15.7%) and includes a 47-bp repeat (∼3.6 copies, positions 523–690 bp; Figure 7, Table 4). This region also contains GA blocks, 5 poly-T stretches, [TA(A)]_n_ blocks, and multiple hairpin structures, with *ΔG* values ranging from –416.7 to –415.4 kcal/mol (Supplementary Figure S4).

### Selection pressure analysis of mitochondrial PCGs

The K_a_/K_s_ ratios in all mitochondrial PCGs showed values <1, indicating that all these PCGs are evolving under an overall purifying selection. Examination of K_a_/K_s_ ratio values in sliding windows across the length of each PCG sequence further indicated that purifying selection is acting along the entire PCG sequence. Analysis of selective pressure on mitochondrial protein-coding genes (PCGs) across different habitat depths revealed that the highest K_a_/K_s_ values were observed in comparisons between intertidal species. Specifically, the comparison between *P. lanuginosus* and *P. maculosus*, both inhabiting shallow intertidal zones (depths <5 m), showed the greatest values. This was followed by the comparison between the shallow-water species *P. gracilipes* (depth <60 m) and the intertidal species *P. filholi* (depth <2 m), which exhibited moderately elevated values. In contrast, the lowest values were observed in the comparison between the shallow-water species *P. gracilipes* and the deep-water species *P. rathbuni* (depths ranging from 60 to 590 m). In more detail, when compared to the values observed between *P. gracilipes* and *P. rathbuni*, the intertidal species inhabiting shallow intertidal zones exhibited higher values in 7 out of the 13 mitochondrial protein-coding genes (*COX1*, *NAD3*, *NAD2*, *NAD4*, *NAD4L*, *CYTOB*, and *NAD1*). Similarly, in the comparison between *P. gracilipes* (habitat depth <60 m) and the intertidal species *P. filholi* (habitat depth <2 m), elevated values were observed in 6 genes (*NAD3*, *NAD2*, *COX3*, *NAD6*, *CYTOB*, and *NAD1*).

## Discussion

### Comparison and similarity of the resulting mtDNA Anomura phylogenetic tree with previous phylogenetic studies

The phylogenetic topology inferred from 42 anomuran species in this study mostly identical with previous mitochondrial DNA (mtDNA)-based phylogenetic analyses (Tan et al. 2018, 2019; Wang et al. 2019, 2023; Gong et al. 2020; Veldsman et al. 2020; Dong et al. 2023).

Interestingly, the phylogenetic tree reconstructed in this study showed similarity to those of studies that incorporated morphological traits and nuclear genes, although some differences were also observed. Pylochelidae, Munididae, and most genera were recovered as monophyletic, whereas earlier studies reported them as polyphyletic (Tsang et al. 2011; Bracken-Grissom et al. 2013).

These differences may be attributed to variation in taxon sampling among studies. This study included 42 species from 25 genera, whereas Tsang et al. (2011) analyzed 46 species from 33 genera, and Bracken-Grissom et al. (2013) analyzed 137 species from 77 genera. In particular, certain genus such as *Trizocheles* could not be included in this study due to the lack of available mitochondrial genome data; however, they have been shown to form distinct clades in other datasets (Tsang et al. 2011). Therefore, incorporating such missing taxa in future studies may help reconcile differences among studies and contribute to a more accurate reconstruction of the evolutionary history of Anomura.

### Diversity and features of mitochondrial gene order in Anomura

Pancrustacea, which encompasses crustaceans and hexapods, typically possesses a conserved mitochondrial gene order, *cox1-trnL1-trnL2-cox2-trnK-nad3-trnM-trnI-nad2-trnD-atp8-atp6-cox3-trnR-trnN-trnS1-trnE-trnF-nad5-trnH-nad4-nad4L-trnT-nad6-cob-trnS2-trnP-nad1-trnW-trnQ-trnC-trnY,* which is often referred to as the Pancrustacean ground pattern, and has been widely used as a critical marker for inferring evolutionary relationships, including the detection of gene-order rearrangements (Boore 1999; Rubin et al. 2000; Lavrov et al. 2004).

Our results revealed that Anomura possesses a total of 25 distinct gene-order types. Notably, no anomuran species shared the typical Pancrustacean ground pattern, suggesting the occurrence of rapid mitochondrial evolution within the group. Among the families examined, the highest level of gene-order diversity was found in Paguridae, with nine distinct types identified, more than in any other family included in this study. More specifically, while the arrangement of protein-coding genes (PCGs) was generally conserved, extensive rearrangements were observed among tRNA genes. Moreover, the predicted secondary structures of the tRNAs were frequently atypical, with more than half of the 22 tRNAs exhibiting non-canonical forms. The occurrence of such atypical tRNA structures may reflect genome streamlining, phylogenetic signals, functional flexibility, and potential environmental adaptation, offering valuable insights into the evolutionary trajectory of mitochondrial genomes in Anomura (Boore 1999; Hickerson and Cunningham 2000). The result that Paguridae exhibits these characteristics can be inferred for the following reason: it comprises more than 600 described species, making it the most species-rich family within the superfamily Paguroidea. Unlike other families that are often confined to specific habitats, members of Paguridae are distributed across a wide range of environments, from shallow coastal waters to the deep sea. Their diverse ecological strategies, including symbiotic relationships, and extensive morphological variation indicate a history of rapid diversification and adaptive evolution (Sultana et al. 2022). The diversity observed in their mitochondrial gene arrangements further supports this evolutionary pattern. Additionally, the result that the newly obtained gene orders of two Lithodidae species are identical to those of certain Paguridae species provides further support for the phylogenetic inference that places Lithodidae as the sister group to Paguridae, as shown in the phylogenetic tree. Meanwhile, the family Galatheoidea exhibited the second highest level of gene-order diversity, with eight distinct types identified, likely reflecting its extensive divergence history. In contrast, other lineages such as Lithodidae, Sternostylidae, and Lomisidae displayed relatively limited variation, suggesting that both the rate and pattern of mitochondrial gene rearrangements may vary considerably among anomuran lineages. Overall, the high degree of gene-order variability observed in Anomura not only challenges the uniform molecular clock hypothesis proposed by Boore (1999) but also clearly highlights the group's dynamic and diversified mitochondrial evolutionary trajectory.

### Comparison of selection pressure analysis of mitochondrial PCGs

A comparative analysis of Ka /Ks ratios across habitat depths revealed that the highest values were observed in comparisons among intertidal species, followed by comparisons between shallow-water and intertidal species, while the lowest values were observed between shallow- and deep-water species. This pattern may be associated with the highly dynamic and physiologically challenging conditions of the intertidal zone, which, for poikilothermic marine crustaceans, can involve severe temperature fluctuations and unstable oxygen levels (see Table 5). In particular, the relatively elevated K_a_/K_s_ values observed in genes involved in oxidative phosphorylation (e.g. *COX1*, *NAD* subunits, and *CYTOB*) (Table 2) appear to reflect relaxed purifying selection in response to the frequent changes in oxygen concentration, temperature, and salinity that characterize intertidal environments (Li et al. 2021; Ramos et al. 2023; Wen et al. 2022). As these genes play a central role in ATP synthesis via the electron transport chain, their accelerated evolution in intertidal species suggests that natural selection may have favored more efficient or flexible energy metabolism under environmental stress. In contrast, species inhabiting shallow or deep-water environments are generally exposed to more stable abiotic conditions, which may have resulted in strong purifying selection acting to maintain mitochondrial function. The lower K_a_/K_s_ values observed in these comparisons are consistent with the hypothesis that stabilizing selection predominates in less variable habitats, thereby contributing to the conservation of mitochondrial gene function (Da Fonseca et al. 2008). Overall, the depth-related differences in selective pressures observed across protein-coding genes (PCGs) suggest that habitat-driven ecological stress may influence the evolutionary trajectory of mitochondrial genomes in crustaceans. Although further research is needed, these findings imply that intertidal environments may exert stronger selective pressures that promote adaptive changes in mitochondrial genes related to energy metabolism, whereas more stable shallow and deep-sea environments may favor purifying selection. This pattern supports the notion that habitat depth plays a critical role in shaping the evolutionary dynamics of mitochondrial genomes. Moreover, carcinization in Anomura, which has independently occurred multiple times throughout evolutionary history, is typically accompanied by morphological traits such as a rigid exoskeleton, a dorsoventrally flattened body, and increased locomotor activity. A rigid and heavily calcified exoskeleton requires substantial energetic investment for formation and maintenance (Palmer 1992). A dorsoventrally flattened body plan and increased locomotor activity has been shown to correlate with elevated oxygen consumption and metabolic rates in crustaceans (Stancil et al. 2024). Accordingly, these features are likely to impose greater physiological demands, particularly in terms of energy consumption and mobility. Therefore, mitochondrial genes involved in oxidative phosphorylation may play a crucial role in meeting these energetic demands. Recent studies suggest that the evolution of the mitochondrial genome may reflect morphological transformations associated with carcinization. For example, Morrison et al. (2002) and Tsang et al. (2011) reported that mitochondrial gene rearrangements occurred repeatedly in carcinized anomuran lineages, implying a potential functional relationship between mitochondrial genomic plasticity and convergent morphological evolution.

**Table 5.** Environmental parameters across depth categories, including *ΔG* ranges, temperature, oxygen concentration, and pressure, with Anomura-specific values indicated. The crustacean control region (CR) is typically 600–1,000 bp long; thus, CRs under 600 bp were excluded.

**Depth Category**	**Range of *ΔG* value (kcal/mol)**	**Range of *ΔG* value (kcal/mol) in Anomura**	**Temperature (°C)**	**Oxygen (mg/L)**	**Pressure (atm)**	**Reference**
Terrestrial	−103.7 to −101.0	−321.5 to −178.3	Variable	Variable	1	NOAA date unknown
Intertidal (0–30 m)	−167.9 to −104	−416.7 to −60.4	10–25	6–8	1–3	
Shallow (30–200 m)	−138.9 to −73.2	−796.4 to −129.2	8–20	4–7	3–20	
Upper bathyal (200–1000 m)			4–8	2–5	20–100	
Deep sea (>1000 m)	N/A	−19.7 to −17.8 (not Hydrothermal vents)	0–4	1–3	100–600	Van Dover 2021
		−198.7 to −21.9 (Hydrothermal vents)	>2–400	0–0.5	>250–400	

### Divergence times of three newly analyzed anomuran mitogenomes and others

Our time-calibrated phylogeny indicates that Anomura diverged from Brachyura around 233 MYA (Norian), with the earliest anomuran split (∼213 MYA, Rhaetian) occurring in the Hippoidea superfamily, which shares morphological traits with primitive Brachyura. Further branching events include the divergence of Galatheoidea and Pylochelidae around 200 MYA (Sinemurian), followed by diversification of subgroups (e.g. Porcellanidae, Munidopsidae) until ∼161 MYA (Kimmeridgian). Aegloidea, Lomisoidea, and Chirostyloidea diverged around 187 MYA (Toarcian), with Lomisoidea emerging first. The final split among Diogenidae, Coenobitidae, Paguridae, and Lithodidae occurred near 159 MYA (Kimmeridgian), aligning with previous findings. Of particular interest, two Hapalogastrinae species (*H. dentata*, *O. inermis*) appear to have branched off earlier than other lithodids, approximately 38–50 MYA (Eocene), whereas *Pagurus rathbuni* diverged about 32 MYA (Oligocene). This timeline coincides with significant climatic shifts during the Eocene–Oligocene Transition, including Antarctic glaciation, global cooling, sea-level decline, and changes in ocean circulation. Selective pressures arising from these events likely influenced marine taxa, promoting physiological and morphological adaptations (Miller et al. 1991; Coxall et al. 2005; Coxall and Pearson 2007; Dunkley Jones and Pearson 2008; Katz et al. 2008). The result that *P. rathbuni* diverged latest may indirectly suggest an influence of temperature, considering that this species inhabits the deep sea, which is characterized by low temperatures, environmental stability, and low metabolic demands, in line with the Evolutionary Speed Hypothesis (Rensch 1959). According to this hypothesis, temperature and metabolic rate determine the rate of evolution, and organisms living in cold and stable environments such as the deep sea or polar regions tend to have lower metabolic and mutation rates and longer generation times, ultimately resulting in slower molecular evolutionary rates. Indeed, previous studies have reported that tropical fish species show faster mitochondrial gene evolution than cold-water species (Wright et al. 2011). This perspective contributes to understanding the evolutionary trajectory of *P. rathbuni* and further suggests that historical climate changes may have driven lineage-specific evolutionary responses within Anomura.

### Mitochondrial control region and ΔG

Among Anomura, three non-carcinized families – Diogenidae, Paguridae, and Munidopsidae – exhibited a positive correlation between mitochondrial control-region Gibbs free energy (*ΔG*) and habitat depth. In contrast, two carcinized families, Lithodidae and Porcellanidae, showed a negative correlation between *ΔG* and depth. These opposing patterns suggest the presence of distinct adaptive strategies between carcinized and non-carcinized lineages. Non-carcinized anomurans may avoid the abrupt environmental fluctuations of intertidal zones through specific morphological traits, such as the strong abdominal flexion observed in squat lobsters or the use of external shells in hermit crabs, and are primarily exposed to gradual selective pressures associated with increasing depth (Seibel and Drazen 2007). These survival strategies likely optimize energy efficiency under low-oxygen conditions, potentially contributing to the observed low *ΔG* values. In contrast, carcinized anomurans such as Lithodidae and Porcellanidae exhibit more flattened and curved abdominal structures (McLaughlin and Lemaitre 1997; Keiler et al. 2017), which may limit mobility in intertidal environments and necessitate greater metabolic flexibility, potentially associated with lower *ΔG* values. Although carcinized forms are also found in deep-sea habitats, the shallow-water environment is often subject to more abrupt and severe stressors, which may account for divergent *ΔG* patterns along depth gradients across lineages. Meanwhile, Munidopsidae exhibit relatively high *ΔG* values compared to other deep-sea anomurans, likely because they inhabit hydrothermal vent environments (Tables 4–5) characterized by elevated temperatures and high organic input (Lutz and Kennish 1993). Members of this family feed on chemosynthetic bacteria and organic detritus, and some species maintain symbiotic relationships with bacteria on their gills or body surfaces (Xiao et al. 2020). As a result, they may experience fewer constraints related to temperature and energy availability with increasing depth, leading to higher *ΔG* values than those observed in other deep-sea anomuran taxa. In conclusion, this study provides foundational insight suggesting that associations between control-region ΔG and metabolic rates, adaptive strategies, and the presence or absence of carcinization in Anomura ‘plausible’. Nevertheless, to clarify these relationships and quantify their ecological and evolutionary implications, further studies incorporating broader taxonomic sampling and detailed environmental data will be essential.

## Supplementary Material

Supplemental Material

## Data Availability

The mitochondrial genome sequences of *Hapalogaster dentata* (OR523818), *Oedignathus inermis* (OR523819), and *Pagurus rathbuni* (OR523820), along with sequences from 42 other decapod species used in this study, are available on GenBank (NCBI).
