# Supplementary material for: Comparative phylomitogenomic analyses provide insights into adaptation and carcinization in Anomura
Source: Anim Cells Syst (Seoul). 2026 Jan 12;30(1):13–33. doi: 10.1080/19768354.2025.2607863 (PMC12798672; doi:10.1080/19768354.2025.2607863)
Supplement: Supplemental Material [file TACS_A_2607863_SM0512.zip › Supplementary Table 1.pdf]

Name	Direction	Start	End	Start codon	Stop codon	Anticodon
COX1 gene	-	1	1542	ATA	TAA	
tRNA-Cys	+	1550	1618			gca
tRNA-Gln	+	1622	1690			uug
tRNA-Trp	-	1692	1759			uca
12S rRNA	+	1788	2581			
tRNA-Val	+	2580	2649			uac
16S rRNA	+	2649	4031			
ND1 gene	+	4035	4979	ATG	ACT	
tRNA-Pro	+	4986	5052			ugg
tRNA-Tyr	-	6366	6433			gua
tRNA-Ser	-	6497	6564			uga
CYTB gene	-	6621	7751	ATA	TAA	
ND6 gene	-	7738	8259	ATT	TAA	
tRNA-Thr	-	8298	8366			ugu
ND4L gene	+	8377	8679	ATG	TAA	
ND4 gene	+	8673	10013	ATG	TAA	
tRNA-His	+	10014	10080			gug
ND5 gene	+	10099	11810	ATT	TTA	
tRNA-Phe	+	11811	11877			gaa
tRNA-Glu	-	11877	11946			uuc
tRNA-Ser	-	11946	12011			ucu
tRNA-Asn	-	12016	12083			guu
tRNA-Arg	-	12083	12145			ucg
COX3 gene	-	12173	12964	ATG	TAA	
ATP6 gene	-	12964	13638	ATG	TAA	
ATP8 gene	-	13632	13790	ATT	TAA	
ND2 gene	-	13848	14858	ATT	TAA	
tRNA-Asp	-	14948	15012			guc
tRNA-Met	-	15027	15094			cau
tRNA-Ile	-	15096	15162			gau
tRNA-Ala	-	15174	15239			ugc
ND3 gene	-	15249	15602	ATT	TAG	
tRNA-Gly	-	15603	15667			ucc
tRNA-Lys	-	15669	15733			uuu
COX2 gene	-	15745	16434	ATG	TAA	
tRNA-Leu	-	16460	16527			uaa
tRNA-Leu	-	16529	16596			uag

Name	Direction	Start	End	Start codon	Stop codon	Anticodon
COX1 gene	-	1	1542	ATA	TAA	
tRNA-Cys	+	1550	1618			gca
tRNA-Gln	+	1622	1690			uug
tRNA-Trp	-	1692	1759			uca
12S rRNA	+	1789	2579			
tRNA-Val	+	2578	2647			uac
16S rRNA	+	2648	3936			
ND1 gene	+	4038	4982	ATG	ACT	
tRNA-Pro	+	4990	5056			ugg
tRNA-Tyr	-	6357	6424			gua
tRNA-Ser	-	6453	6521			uga
CYTB gene	-	6591	7721	ATA	TAA	
ND6 gene	-	7708	8229	ATC	TAA	
tRNA-Thr	-	8274	8343			ugu
ND4L gene	+	8353	8655	ATG	TAA	
ND4 gene	+	8649	9977	ATG	ATG	
tRNA-His	+	9990	10056			gug
ND5 gene	+	10075	11786	ATT	CAA	
tRNA-Phe	+	11787	11854			gaa
tRNA-Glu	-	11853	11922			uuc
tRNA-Ser	-	11922	11987			ucu
tRNA-Asn	-	11993	12061			guu
tRNA-Arg	-	12061	12123			ucg
COX3 gene	-	12151	12942	ATG	TAA	
ATP6 gene	-	12942	13616	ATG	TAA	
ATP8 gene	-	13610	13768	ATT	TAA	
ND2 gene	-	13823	14833	ATT	TAA	
tRNA-Asp	-	14921	14986			guc
tRNA-Met	-	15009	15076			cau
tRNA-Ile	-	15077	15143			gau
tRNA-Ala	-	15155	15220			ugc
ND3 gene	-	15230	15583	ATT	TAG	
tRNA-Gly	-	15584	15649			ucc
tRNA-Lys	-	15650	15715			uuu
COX2 gene	-	15727	16416	ATG	TAA	
tRNA-Leu	-	16441	16508			uaa
tRNA-Leu	-	16510	16574			uag

Name	Direction	Start	End	Start codon	Stop codon	Anticodon
COX1 gene	+	1	1503	ATT	TAA	
tRNA-Leu	+	1515	1581			uag
tRNA-Leu	+	1583	1650			uaa
COX2 gene	+	1684	2373	ATG	TAA	
tRNA-Lys	+	2384	2456			uuu
tRNA-Gly	+	2457	2521			ucc
ND3 gene	+	2522	2875	ATT	TAA	
tRNA-Ala	+	2885	2950			ugc
tRNA-Ile	+	2962	3028			gau
tRNA-Met	+	3029	3096			cau
tRNA-Asp	+	3116	3180			guc
ND2 gene	+	3251	4291	ATC	TAA	
ATP8 gene	+	4346	4504	ATT	TAA	
ATP6 gene	+	4498	5172	ATG	TAA	
COX3 gene	+	5172	5963	ATG	TAG	
tRNA-Arg	+	5991	6054			ucg
tRNA-Asn	+	6054	6121			guu
tRNA-Ser	+	6128	6193			ucu
tRNA-Glu	+	6193	6262			uuc
tRNA-Phe	-	6261	6326			gaa
ND5 gene	-	6327	8038	ATT	TTA	
tRNA-His	-	8057	8123			gug
ND4 gene	-	8136	9464	ATG	TAA	
ND4L gene	-	9458	9760	ATG	TAA	
tRNA-Thr	+	9767	9835			ugu
ND6 gene	+	9866	10405	ATT	TAA	
CYTB gene	+	10374	11504	ATA	TAA	
tRNA-Ser	+	11602	11668			uga
tRNA-Tyr	+	11736	11803			gua
tRNA-Pro	-	12928	12993			ugg
ND1 gene	-	13000	13944	ATG	ATT	
16S rRNA	-	13962	15322			
tRNA-Val	-	15323	15392			uac
12S rRNA	-	15391	16182			
tRNA-Trp	+	16213	16280			uca
tRNA-Gln	-	16282	16350			uug
tRNA-Cys	-	16354	16420			gca
