# Supplementary material for: Comparative phylomitogenomic analyses provide insights into adaptation and carcinization in Anomura
Source: Anim Cells Syst (Seoul). 2026 Jan 12;30(1):13–33. doi: 10.1080/19768354.2025.2607863 (PMC12798672; doi:10.1080/19768354.2025.2607863)
Supplement: Supplemental Material [file TACS_A_2607863_SM0512.zip › Supplementary_Figure_5_second.pdf]

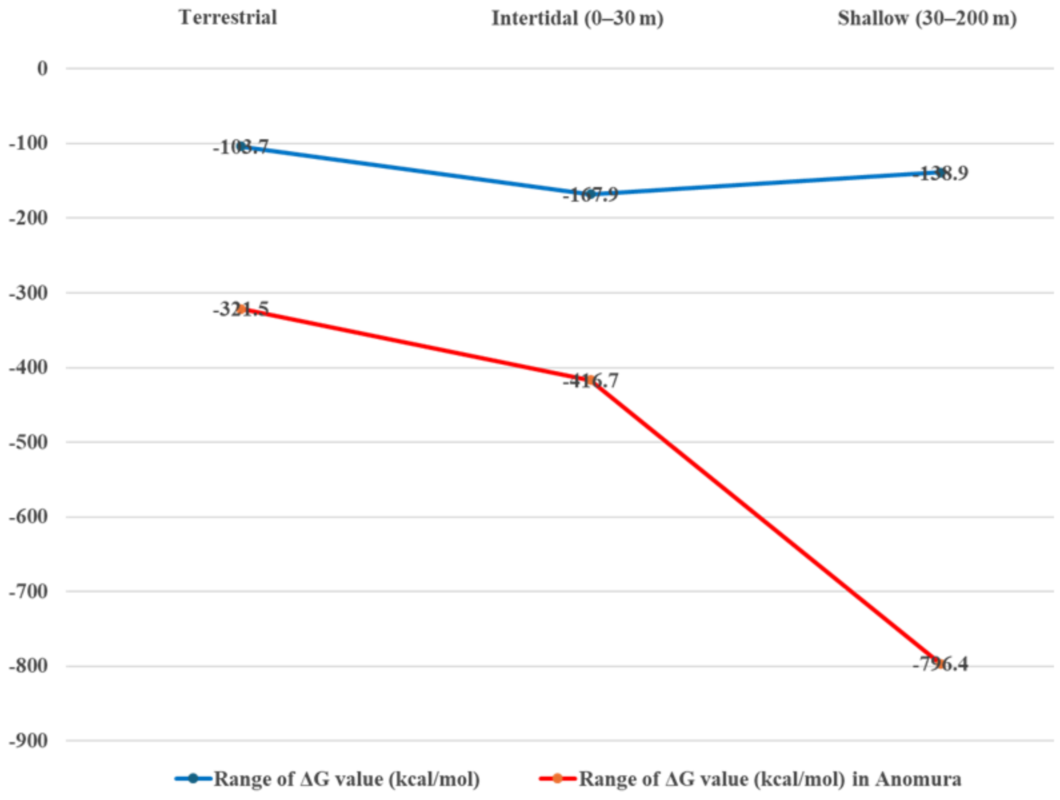
Terrestrial
Intertidal (0–30 m)
Shallow (30–200 m)
0
-100
-200
-300
-400
-500
-600
-700
-800
-900
-103.7
-167.9
-138.9
-321.5
-416.7
-796.4
Range of ΔG value (kcal/mol)
Range of ΔG value (kcal/mol) in Anomura
