# Supplementary figures and images for: Comparative phylomitogenomic analyses provide insights into adaptation and carcinization in Anomura
Source: Anim Cells Syst (Seoul). 2026 Jan 12;30(1):13–33. doi: 10.1080/19768354.2025.2607863 (PMC12798672; doi:10.1080/19768354.2025.2607863)

## *Pagurus rathbuni*

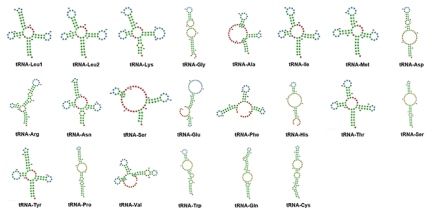

## *Oedignathus inermis*

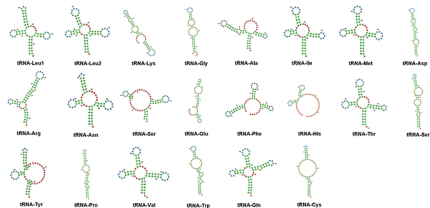

## *Hapalogaster dentata*

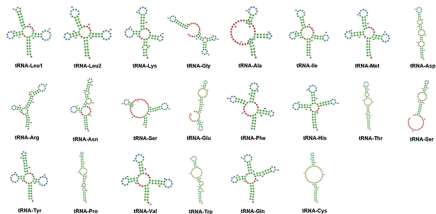

Supplement: Supplemental Material [file TACS_A_2607863_SM0512.zip › Supplementary_Figure_1_revised.pdf]

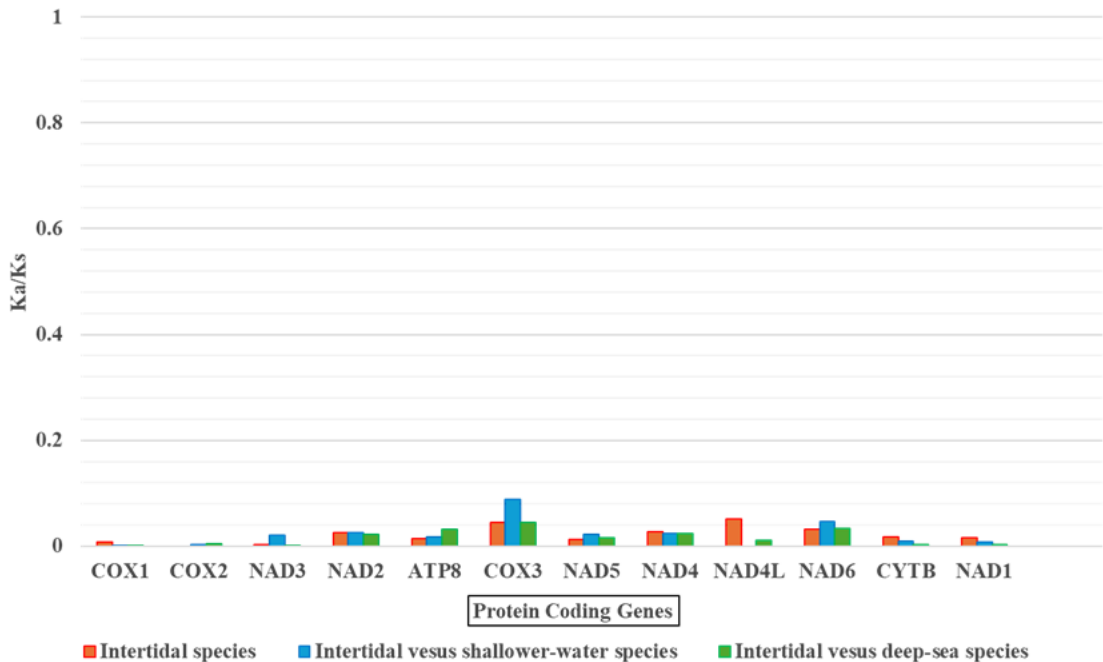

Supplement: Supplemental Material [file TACS_A_2607863_SM0512.zip › Supplementary_Figure_6_second.pdf]
